# Supplementary material for: Proteobacteria Overgrowth and Butyrate-Producing Taxa Depletion in the Gut Microbiota of Glycogen Storage Disease Type 1 Patients
Source: Metabolites. 2020 Mar 30;10(4):133. doi: 10.3390/metabo10040133 (PMC7240959; doi:10.3390/metabo10040133)
Supplement: Supplementary file 1 [file metabolites-10-00133-s001.pdf]

Supplementary Materials

Table 1. Blood biochemical parameters in GSD patients. Values are expressed as mean (standard deviation). Reference values are reported.

	GSD Mean (SD)	GSD Ia Mean (SD)	GSD Ib Mean (SD)	Reference values
**Glucose, fasting**	71.38 (10.28)	65.50 (7.05)	80.00 (10.79)	70–105 mg/dL
**Insulin, fasting**	5.88 (9.74)	2.45 (2.86)	8.96 (11.70)	0-25 uU/ml
**HOMA-IR**	1.10 (1.89)	0.42 (0.54)	1.76 (2.26)	
**QUICKI Index**	0.47 (0.12)	0.53 (0.13)	0.39 (0.07)	
**HOMA-β**	1.64 (2.59)	0.76 (0.77)	2.37 (3.14)	
**Tyg-Index**	3.95 (0.42)	4.18 (0.39)	3.80 (0.35)	
**Cholesterol, total**	206.75 (124.68)	265.50 (152.17)	166.40 (68.02)	< 200 mg/dl
**Triglycerides**	300.25 (225.92)	422.50 (241.58)	204.60 (138.56)	<150 mg/dL
**Uric acid**	6.675 (1.03)	7.10 (0.50)	6.25 (3.02)	2.5–8 mg/dL
**Serum lactate**	3.00 (1.77)	3.85 (1.93)	2.14 (1.10)	0.7-2.1 mmol/L
**AST**	42.5 (23.84)	54.50 (28.35)	37.80 (19.25)	0–35 U/L
**ALT**	54.13 (43.44)	67.75 (47.13)	50.80 (42.36)	0–35 U/L

Table 2. Fecal SCFA concentrations. For each experimental group, mean and standard deviation from Mann-Whitney test are reported. Values are expressed as mean (standard deviation). P-values <0.05 were considered significant.

	HC<br>Mean (SD)	GSD<br>Mean (SD)	p-value
**Acetate**	1.84 (0.73)	3.54 (1.95)	0.0310*
**Propionate**	0.72 (0.24)	1.41 (1.07)	0.0381*
***iso*-butyrate**	0.29 (0.12)	0.28 (0.22)	0.8965
**Butyrate**	0.32 (0.12)	0.33 (0.22)	0.8381
***iso*-valerate**	0.24 (0.11)	0.19 (0.16)	0.3598
**Total SCFAs**	3.40 (0.85)	5.75 (2.88)	0.0159*
